# Vision-Based Instance Segmentation of Piezoelectric Crystal Defects Using a Hybrid Bidirectional CNN–Transformer Network

**DOI:** 10.3390/s26175480

**Published:** 2026-08-29

**Authors:** Zeyao Hou, Zongyu He, Haotian Huang, Xiaoyan Chen

**Affiliations:** 1 College of Mechanical Engineering, Tianjin University of Science and Technology, Tianjin 300222, China; 2 College of Low-Altitude Systems and Engineering, Tianjin College, University of Science and Technology Beijing, Tianjin 301830, China

**Keywords:** piezoelectric crystal, defect segmentation, instance segmentation, CNN–Transformer network, multi-scale feature fusion, industrial inspection

## Abstract

Accurate defect instance segmentation is essential for automated quality inspection of piezoelectric crystals, where scratches, stains, and other surface defects often show large scale variation, irregular morphology, and weak contrast against complex backgrounds. Existing CNN-based segmentation models capture local texture effectively but are limited in global context modeling, whereas Transformer-based models often require stronger local detail preservation for fine industrial defects. To address these limitations, this paper proposes a hybrid bidirectional bridging CNN–Transformer network, named HBCTNet, for piezoelectric crystal defect instance segmentation. HBCTNet introduces a Convolution-Transformer Bidirectional Bridging Block (CTB) that enables lightweight two-way interaction between convolutional local features and Transformer global representations. A Frequency and Spatial Convolution (FSC) module is designed to enhance defect-related details in both spatial and frequency domains, and a Multi-Scale Feature Pyramid Network (MS-FPN) fuses features across scales. On the reported image partition of the self-constructed dataset, HBCTNet-S obtains 92.3% box AP and 89.9% mask AP with 12.67 M parameters and 48.1 G FLOPs. Its mask AP is 2.1 percentage points higher than YOLO11-SEG and 2.4 percentage points higher than YOLOv9-SEG under the reported comparison. These results support the effectiveness of the architecture within the evaluated dataset; inference speed and cross-domain generalization remain to be assessed.

## 1. Introduction

Industrial production is a key foundation of modern manufacturing, and reliable product quality inspection is essential for stable process control [1]. Piezoelectric/quartz crystals are widely used in high-precision applications, including medical devices, aerospace systems, automotive electronics, and telecommunications. Surface defects such as scratches, stains, and local structural damage may degrade product reliability and therefore must be detected accurately and efficiently. Deep learning has been increasingly introduced into industrial defect inspection and has achieved promising results [2,3,4]. However, practical inspection scenarios remain challenging because defects usually exhibit irregular shapes, diverse categories, weak contrast, and complex spatial distributions. This study therefore focuses on accurate instance segmentation of piezoelectric crystal defects in industrial production lines, with the aim of improving both inspection accuracy and efficiency [1,2,4].

Early deep-learning-based inspection methods mainly relied on classification networks to determine whether a defect was present. AlexNet [5] introduced convolutional neural networks (CNNs) for image feature extraction and substantially improved classification accuracy and speed over traditional methods. Subsequent networks, including VGG [6], GoogLeNet [7], ResNet [8], CSPNet [9], and GhostNet [10], further improved feature extraction and classification performance [5,6,7,8,9,10].

Because industrial defects are often irregular and spatially complex, instance segmentation is more suitable than image-level classification for many inspection tasks. It provides pixel-level localization of each defect and supports subsequent defect assessment and process analysis. Representative instance segmentation methods include Mask R-CNN [11], ShapeMask [12], YOLACT [13], TensorMask [14], RetinaMask [15], and segmentation variants of YOLOv5, YOLOv7 [16], YOLOv8, YOLOv9 [17], and YOLO11. Recent studies have also adapted these models to industrial inspection. Wu et al. [18] embedded the scSE attention mechanism into a MobileNetV2 backbone, improved the FPN module, and designed a Restrained-IoU bounding-box loss. He et al. [19] proposed TFDS-YOLOv8n for defect segmentation by introducing coordinate attention, Slim-Neck feature fusion, and MPDIoU loss. Zhang et al. [20] developed YOLOv8-CM for detecting and segmenting tunnel defects and key objects. Hybrid YOLO-Swin Transformer methods have also been shown to improve surface-defect detection accuracy [11,12,13,14,15,16,17,18,19,20,21].

The Transformer architecture has recently become an important direction in computer vision. Dosovitskiy et al. [22] proposed the Vision Transformer (ViT), which demonstrated the potential of self-attention for image classification and helped transfer Transformer modeling from natural language processing to visual tasks. Transformer-based models have since been applied to image classification [23], object detection [24], semantic segmentation [25,26], and video understanding [27]. Carion et al. [28] proposed DETR, an end-to-end Transformer-based object detection framework. Liu et al. [29] introduced the Swin Transformer, a hierarchical architecture using shifted windows for efficient dense prediction. Srinivas et al. [30] proposed BoTNet, which replaces selected convolutional layers with multi-head self-attention and achieves strong visual recognition performance 3×3 [22,23,24,25,26,27,28,29,30].

CNNs are effective at extracting local features such as edges, textures, and shapes through local receptive fields, weight sharing, and spatial inductive bias. Transformers, in contrast, are well suited to modeling long-range dependencies and global context. As visual tasks increasingly require both local detail and global reasoning, hybrid CNN–Transformer architectures have become a promising solution. Such architectures combine the local representation capability of CNNs with the global modeling capability of Transformers to produce stronger visual features [31,32].

A common strategy is to embed Transformer modules into conventional CNN architectures to introduce global feature extraction. BoTNet inserts Transformer modules into ResNet and verifies the effectiveness of this design for image classification. CoAtNet [33] combines EfficientNet-style convolutional feature extraction with attention mechanisms in a hierarchical architecture. ConViT [34] introduces soft convolutional inductive biases into Transformers to strengthen local perception. However, many existing CNN–Transformer combinations use simple serial connections, which may be insufficient for deep interaction between local and global features [33,34].

Parallel CNN–Transformer designs have also been explored. Liu et al. [35] combined CNN and Swin Transformer modules into an efficient parallel structure that integrates local and global modeling. Wu et al. [36] designed a hybrid CNN–Transformer network using multi-scale residual encoding and Transformer-based decoding to learn and fuse local and global contextual information [35,36].

Beyond stacking, serial connection, and parallel design, bridging mechanisms have been introduced to improve cross-branch feature interaction. Mobile-Former connects a MobileNet branch to a small set of learned global tokens through lightweight bidirectional cross-attention [37]. ViT-CoMer instead retains independent CNN and plain-ViT branches and applies multi-stage CNN–Transformer interaction together with a multi-receptive-field feature pyramid [38]. HBCTNet does not claim to introduce bidirectional CNN–Transformer interaction itself. Its distinction is a stage-adaptive block that splits a same-resolution feature map by channel, combines an FSC local branch with a CGT context branch, and exchanges their features through reciprocal 3 × 3 projections before 1 × 1 fusion. This organization differs from the learned-token bridge of Mobile-Former and the independent full CNN/ViT branches of ViT-CoMer. Other architectures, including SpikingResformer [39], CAF-Former [40], and CTC-Net [41], further demonstrate the value of local–global interaction in different application domains.

Accordingly, the principal methodological contribution of HBCTNet is not a generic bidirectional bridge, but the coupling of FSC-based spatial-frequency detail extraction with a stage-adaptive CGT branch and a lightweight reciprocal projection inside the backbone. MS-FPN then aligns and aggregates the last three backbone scales using MSF and AHDown. The reported experiments evaluate this task-specific combination on the self-constructed piezoelectric crystal dataset.

The main contributions of this study are as follows:

1. A stage-adaptive HBCTNet architecture is proposed for piezoelectric crystal defect instance segmentation. In the selected configuration, the first two backbone stages operate as FSC-only stages, while the deeper stages activate the proposed Convolution-Transformer Bidirectional Bridging Block (CTB) according to the selected branch ratios described in Section 3.2.4.
2. The CTB couples an FSC-based convolutional branch and a CGT-based Transformer branch through two reciprocal 3 × 3 channel projections. The design performs one explicit CNN-to-Transformer and one Transformer-to-CNN exchange before concatenation and 1 × 1 output fusion.
3. An MS-FPN is constructed around a task-specific MSF-AHDown pathway: C3–C5 features are aligned, combined by concatenation, and processed by parallel depthwise kernels whose responses are combined by element-wise summation before pointwise projection and residual fusion. The contribution is this arrangement rather than the general concept of a feature pyramid.
4. On the reported image partition of the self-constructed dataset, HBCTNet-S reaches 92.3% box AP and 89.9% mask AP with 12.67 M parameters and 48.1 G FLOPs. Cross-domain performance, run-to-run variability, and deployment latency are outside the evidence provided by the present experiments.

The remainder of this paper is organized as follows: Section 2 presents the architecture of HBCTNet and its main modules. Section 3 describes the dataset, implementation details, ablation studies, and comparison experiments. Section 4 discusses the experimental findings and practical implications. Section 5 concludes the paper and outlines future work.

## 2. Methodology

This section describes the overall architecture of HBCTNet and its key components, including the Frequency and Spatial Convolution (FSC) module, the Convolutional Gated Linear Unit Vision Transformer (CGT) module, the Convolution-Transformer Bidirectional Bridging Block (CTB), the Multi-Scale Feature Pyramid Network (MS-FPN), the Multi-Scale Feature Fusion (MSF) module, and the AvgPool Haar Down (AHDown) module.

**Figure 1:**
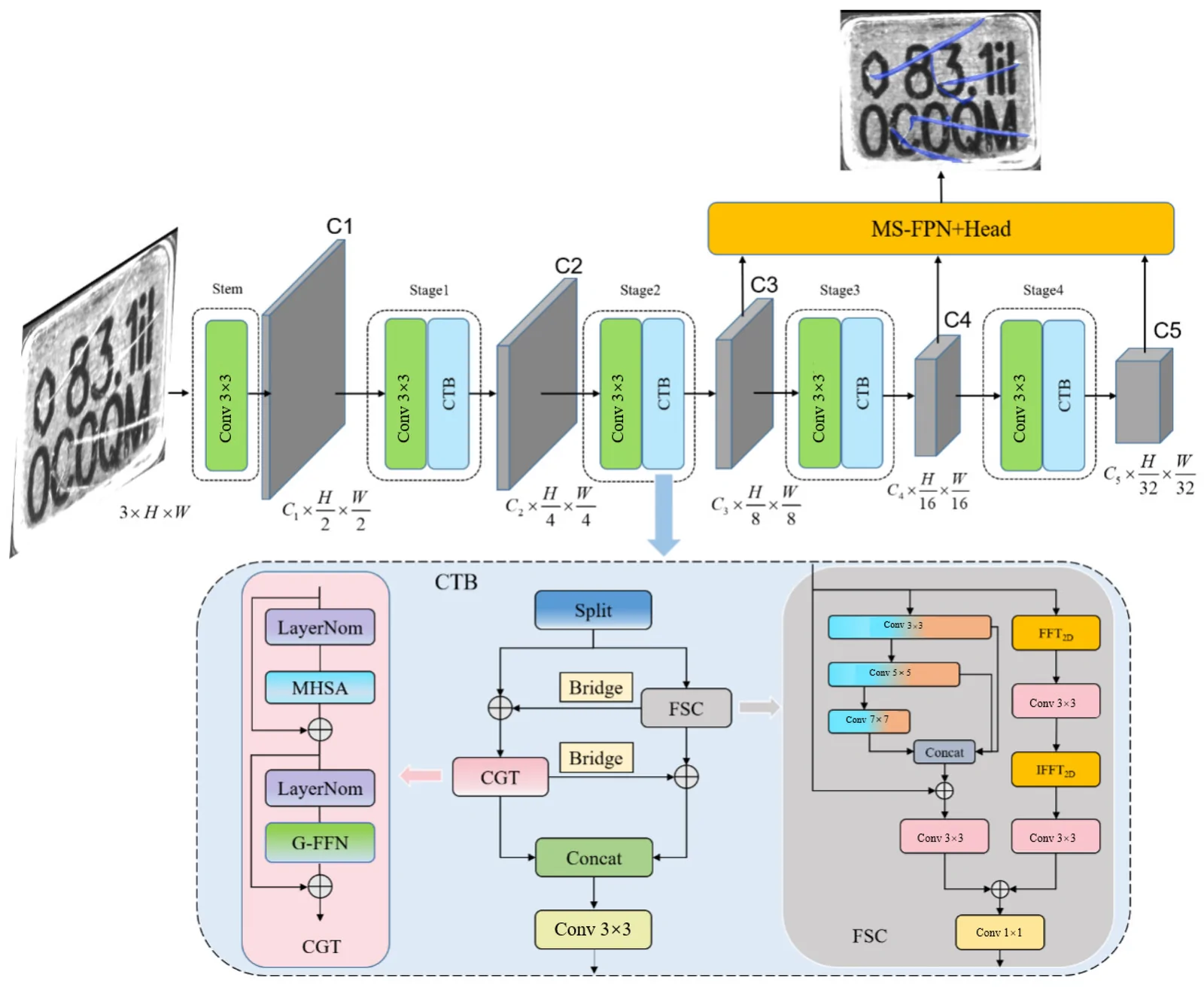
Overall architecture of HBCTNet. The CTB icon denotes the generic stage template. In the selected configuration, Stages 1–2 use FSC only because the Transformer coefficient is zero, whereas Stages 3–4 activate the bidirectional FSC-CGT branches; the exact ratios are described in Section 3.2.4.

As shown in Figure 1, HBCTNet adopts a hierarchical architecture. The stage drawing represents a generic FSC-CGT template, while the active branches are determined by the selected branch configuration. In the selected HBCTNet-S configuration, Stages 1 and 2 use coefficients (1, 0) and therefore operate without a Transformer branch; Stages 3 and 4 use (0.75, 0.25) and (0.5, 0.5), respectively, and activate bidirectional local–global interaction. Strided convolutions are used for downsampling, and the network produces feature maps at 8×, 16×, and 32× downsampling ratios for MS-FPN.

### 2.1. Frequency and Spatial Convolution (FSC)

The proposed FSC module extracts image features in both the spatial and frequency domains. By jointly modeling spatial structures and frequency components, FSC enhances details, edges, and texture-related information, thereby improving adaptability to industrial images with diverse defect patterns.

#### 2.1.1. Spatial-Domain Branch

The spatial-domain branch is inspired by GhostNet and FasterNet. In FasterNet, partial convolution (PConv) applies conventional convolution to only part of the input channels while leaving the remaining channels unchanged [42]. This design reduces computation while preserving valid spatial information. GhostNet generates additional “ghost” features from a smaller set of intrinsic features, enriching representation with limited computational overhead [42].

**Figure 2:**
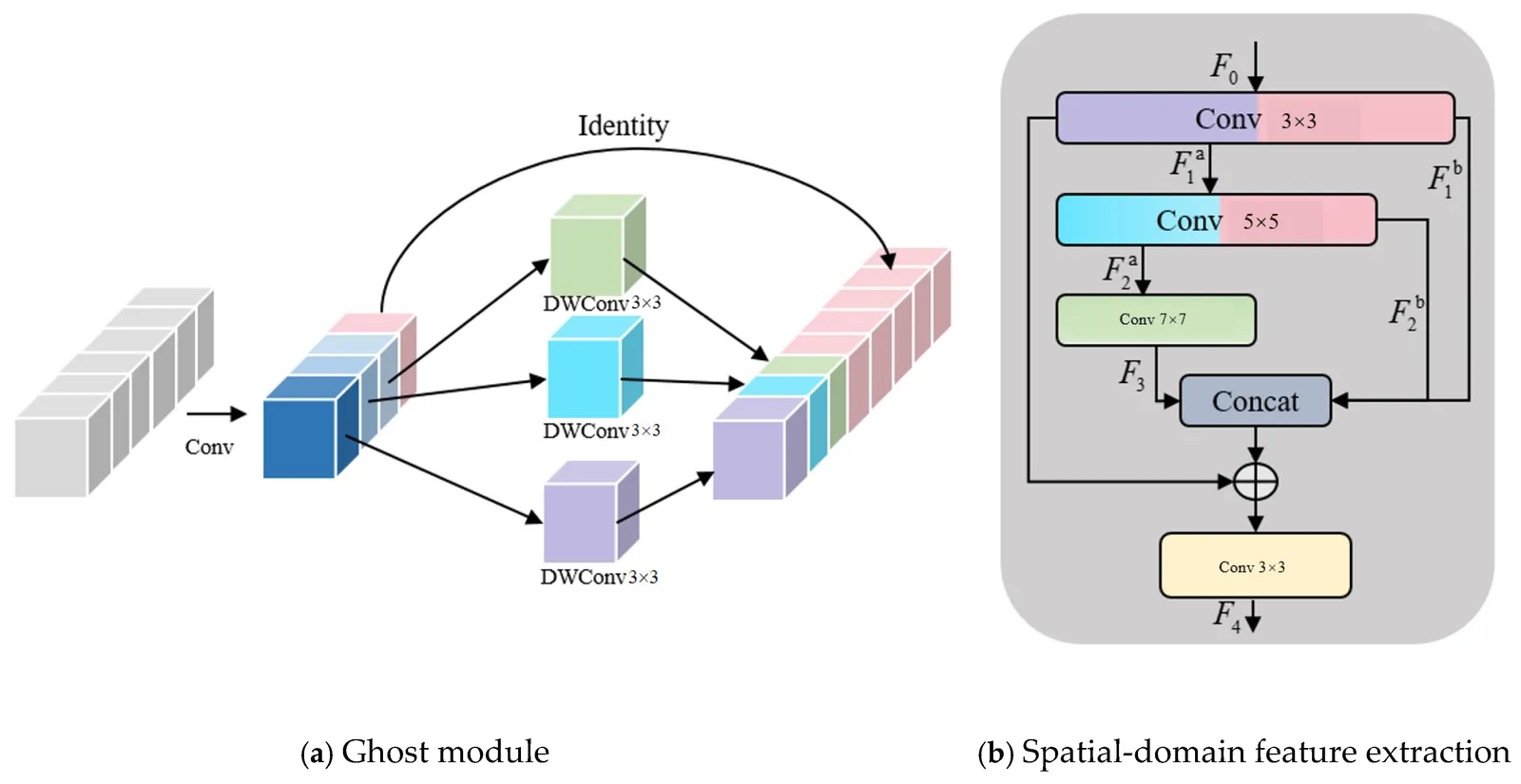
(**a**) Structure of the Ghost module, where Identity denotes no operation. (**b**) Spatial-domain feature extraction in the FSC module. The pink part represents features that bypass convolution and are directly fused downward.

As shown in Figure 2b, the spatial-domain feature extraction branch first applies a convolution to the input feature. Following the idea of partial convolution, the feature is split into two channel groups, and convolution is applied only to the selected group. The same process is then repeated progressively. Compared with applying convolution to all channels, this progressive channel-selective strategy reduces computational cost and improves feature extraction efficiency. The final output can be expressed as follows: 3×3 $F_{0}\in {\mathbb{R}}^{H\times W\times C}$ $F_{1}\in {\mathbb{R}}^{H\times W\times C}$ $F_{1}$ $F_{1}^{a}\in {\mathbb{R}}^{H\times W\times \frac{C}{2}}$ $F_{1}^{b}\in {\mathbb{R}}^{H\times W\times \frac{C}{2}}$ $F_{1}^{a}$ 5×5 $F_{2}\in {\mathbb{R}}^{H\times W\times \frac{C}{2}}$ $F_{2}$ $F_{2}^{a}\in {\mathbb{R}}^{H\times W\times \frac{C}{4}}$ $F_{2}^{b}\in {\mathbb{R}}^{H\times W\times \frac{C}{4}}$ 7×7 $F_{2}^{a}$ $F_{3}\in {\mathbb{R}}^{H\times W\times \frac{C}{4}}$ $F_{4}\in {\mathbb{R}}^{H\times W\times C}$.

(1) $$F_{4}=C\mathrm{onv}(F_{0}+C\mathrm{oncat}(F_{1}^{b},F_{2}^{b},F_{3}))$$

Three convolution kernels with different sizes are used to extract multi-scale spatial information, which improves the model’s ability to recognize and segment defects in complex scenes.

#### 2.1.2. Frequency-Domain Branch

**Figure 3:**
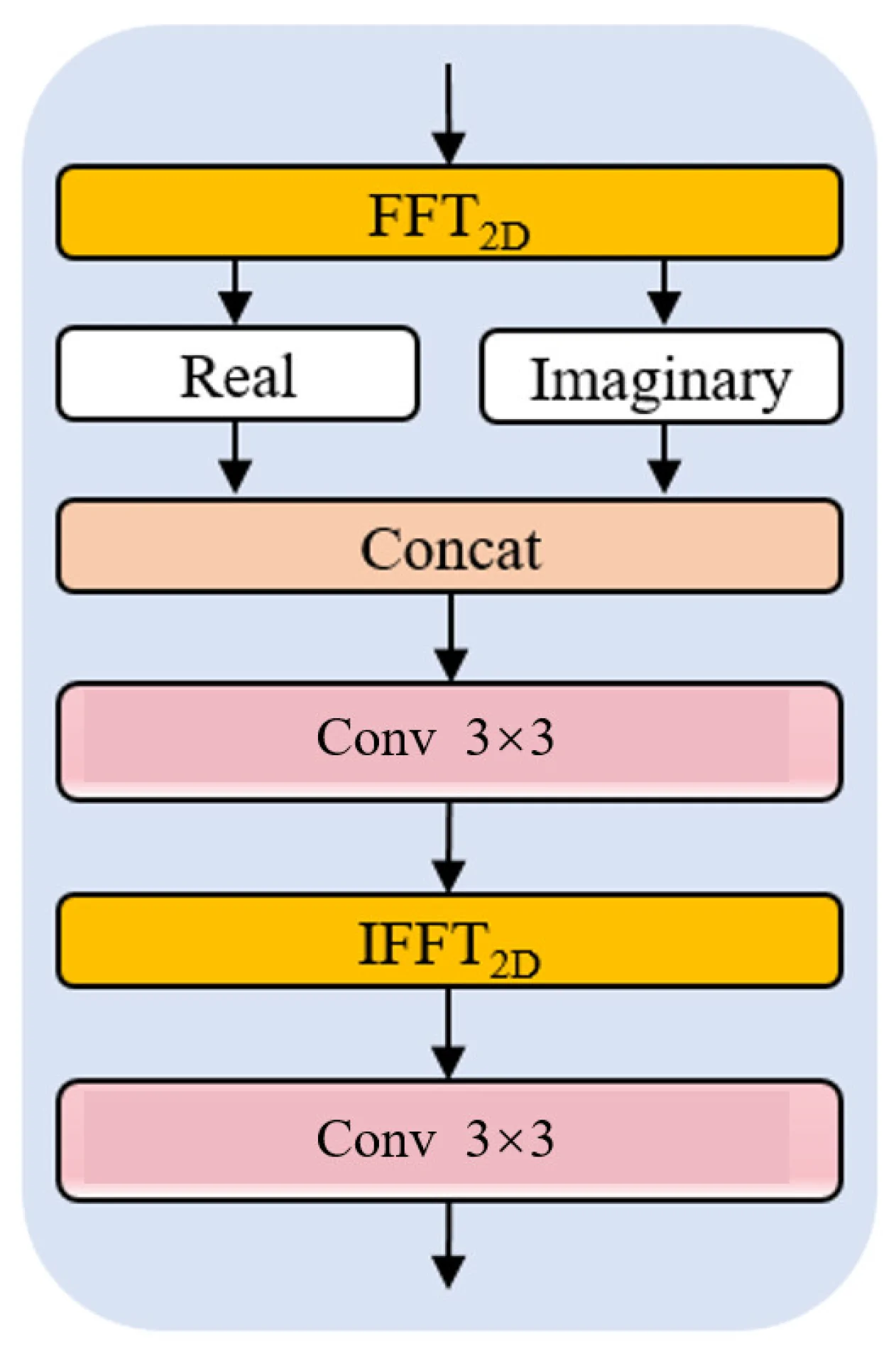
Structure of the frequency-domain feature extraction branch in the FSC module.

As shown in Figure 3, frequency-domain feature extraction uses the Fourier transform to convert an image from the spatial domain to the frequency domain. Different frequency components describe image structures at different spatial scales. Low-frequency components contain global structure and large-scale information, whereas high-frequency components contain details, edges, and noise. Frequency-domain modeling is therefore useful for capturing both global and local information, especially for textures, repetitive patterns, and periodic structures [43,44].

In the frequency-domain feature extraction section, we first use a two-dimensional Fast Fourier Transform (FFT) to convert the image from the time domain to the frequency domain:

(2) $$\hat{X}(f)=FFT_{2D}(x)$$

where the transformed representation contains amplitude and phase information associated with specific frequency components, allowing the network to model periodic structure and repetitive texture features $\hat{X}(f)$.

We assume a two-dimensional image x with dimensions M × N:

(3) x={x[m,n]},m=0,1,2,…,M−1,n=0,1,2,…,N−1

x[m,n] represents the value of each pixel in the image. Therefore, the Fast Fourier Transform (FFT) formula can be written as:

(4) $$X(k,l)=\sum_{m=0}^{M-1}\sum_{n=0}^{N-1}x(m,n)e^{-j2\pi (\frac{km}{M}+\frac{\ln}{N})}$$

Here, we perform a weighted summation for each pixel x[m,n] to obtain its corresponding frequency component X(k,l) in the frequency domain. The exponential term represents the contribution of each pixel to different frequencies in the frequency domain.

Then, we separate the real and imaginary parts of the frequency-domain image, resulting in two feature maps, each representing different frequency components of the image. These frequency components are processed using frequency-domain convolution, allowing us to extract more complex and higher-order frequency features. Finally, we perform an inverse Fourier transform to map the results back to the spatial domain:

(5) $$\hat{X}=IFFT_{2D}(Conv(Concat({\hat{X}}_{\mathrm{real}},\hat{X_{\mathrm{imag}}})))$$

In this case, X_real and X_imag represent the real and imaginary parts of the image in the frequency domain, respectively:

(6) X^real=Re(X^(f)),X^imag=Im(X^(f))

Here, we use the following formula for the inverse Fourier transform:

x[m,n] = (1/(MN)) Σ(k=0 to M−1) Σ(l=0 to N−1) X(k,l) exp[j2π(km/M + ln/N)](7)

The coefficient 1/(MN) in the inverse transform formula is a normalization factor, which ensures the reversibility of the transformation and guarantees that the spatial-domain image reconstructed from the frequency domain is consistent with the original image.

### 2.2. Convolutional Gated Linear Unit Vision Transformer (CGT) Module

The CGT module receives the Transformer-branch feature only in stages where that branch is active. For a stage-s input $X_{t}$ with shape $B\times C_{t}\times H_{s}\times W_{s}$, convolutional projections generate Q, K, and V. The spatial dimensions are flattened to $N_{s}=H_{s}W_{s}$ tokens and rearranged to $B\times h\times N_{s}\times d_{k}$, where h is the number of heads and $d_{k}=C_{t}/h$. In the formulation of Equation (8), scaled dot-product attention is calculated independently in each head over all N_s_ spatial positions; the head outputs are concatenated and linearly projected. The calculation is defined as follows:

(8) $$\mathrm{Attention}(Q,K,V)=\mathrm{softmax}(\frac{QK^{T}}{\sqrt{d_{k}}})V$$

Here, Q, K, and V denote the queries, keys, and values, respectively, and $d_{k}$ is the per-head key dimension used in Equation (8). No separate positional-encoding term or restricted attention window is shown in the reported equation; whether either operation is applied elsewhere in the implementation must be verified from the model code. If Equation (8) is applied over all $N_{s}$ positions, the attention score and value aggregation require $O(N_{s}^{2}C_{t})$ operations in addition to the projection cost. Because the selected architecture activates CGT only in the 1/16- and 1/32-resolution stages, this potential quadratic term is confined to the deeper, lower-resolution feature maps.

**Figure 4:**
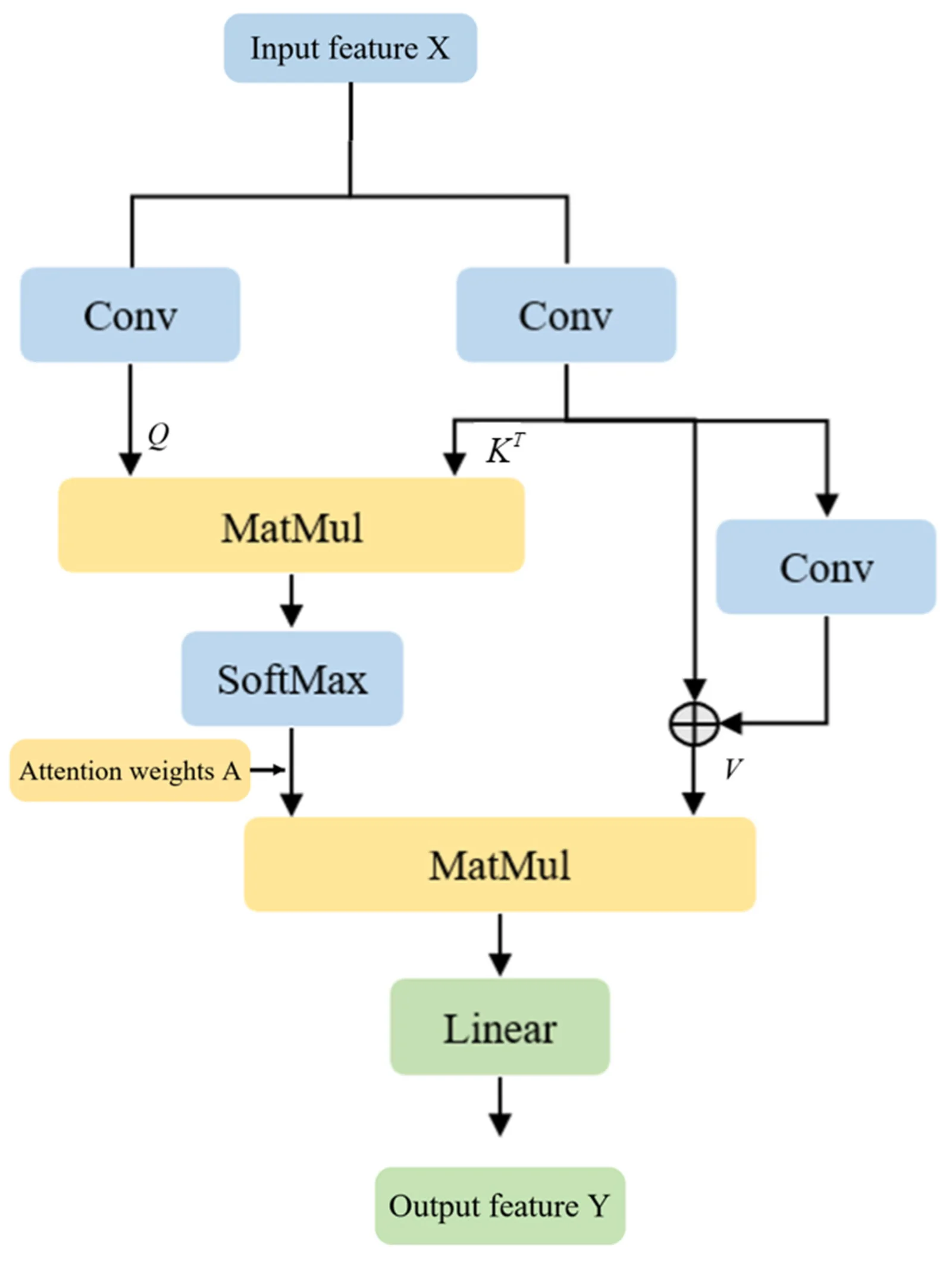
Attention computation for one head. Q and K form the scaled dot-product weight matrix $A=\mathrm{softmax}(QK^{T}/\sqrt{d_{k}})$; A aggregates V, and a linear projection produces the head output. The per-head input and output shapes are $B\times N_{s}\times d_{k}$. Eight structurally identical heads are used in the selected configuration.

We further adopt the idea of the gated linear unit [45] to construct a feed-forward network for ViT, denoted as G-FFN. The feature is divided into two parts by convolution. One part acts as a gating signal, while the other is processed by depthwise separable convolution for feature extraction. The two parts are multiplied element-wise to implement selective feature activation. This mechanism allows the model to preserve useful features and suppress less informative responses, thereby improving nonlinear representation capability. The process is described as follows; the corresponding single-head attention computation is illustrated in Figure 4:

(9) y,v=Conv(X),Y=Conv(y+DWConv(x⊙v))

The input feature is X. Conv denotes the 1 × 1 convolution, DWConv denotes the 3 × 3 depthwise separable convolution, and ⊙ denotes the element-wise multiplication operation.

To alleviate vanishing or exploding gradients and improve training stability, residual connections are introduced after both MHSA and G-FFN. The module output is calculated as follows:

X=DropPath(MHSA(LayerNorm(x)))+x

(10) Output=DropPath(G−FFN(LayerNorm(x)))+X

**Figure 5:**
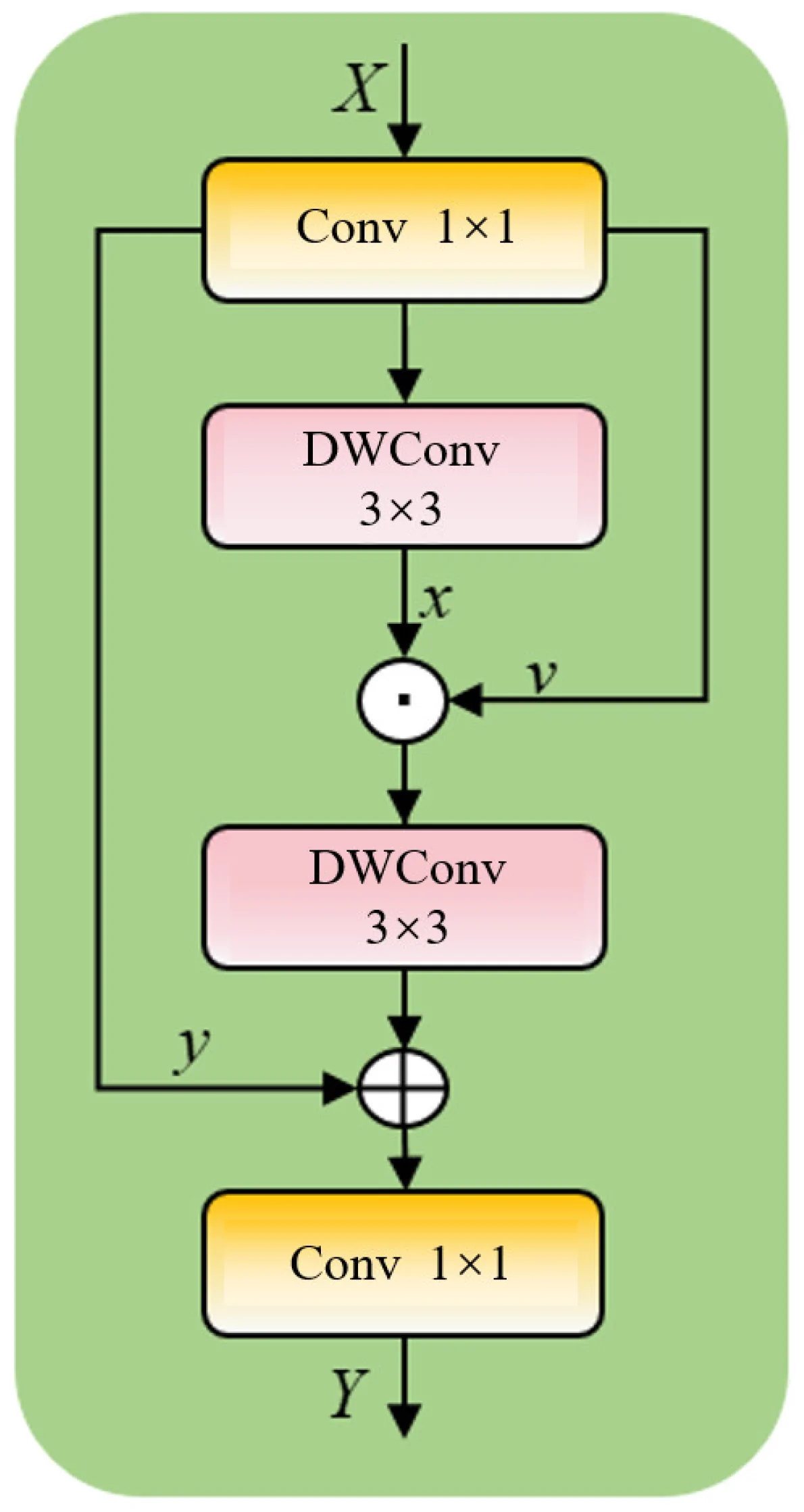
Architecture of the G-FFN.

This residual strategy stabilizes optimization and accelerates convergence by enabling smoother gradient propagation. The architecture of the G-FFN is shown in Figure 5.

### 2.3. Convolution-Transformer Bidirectional Bridging Block (CTB)

**Figure 6:**
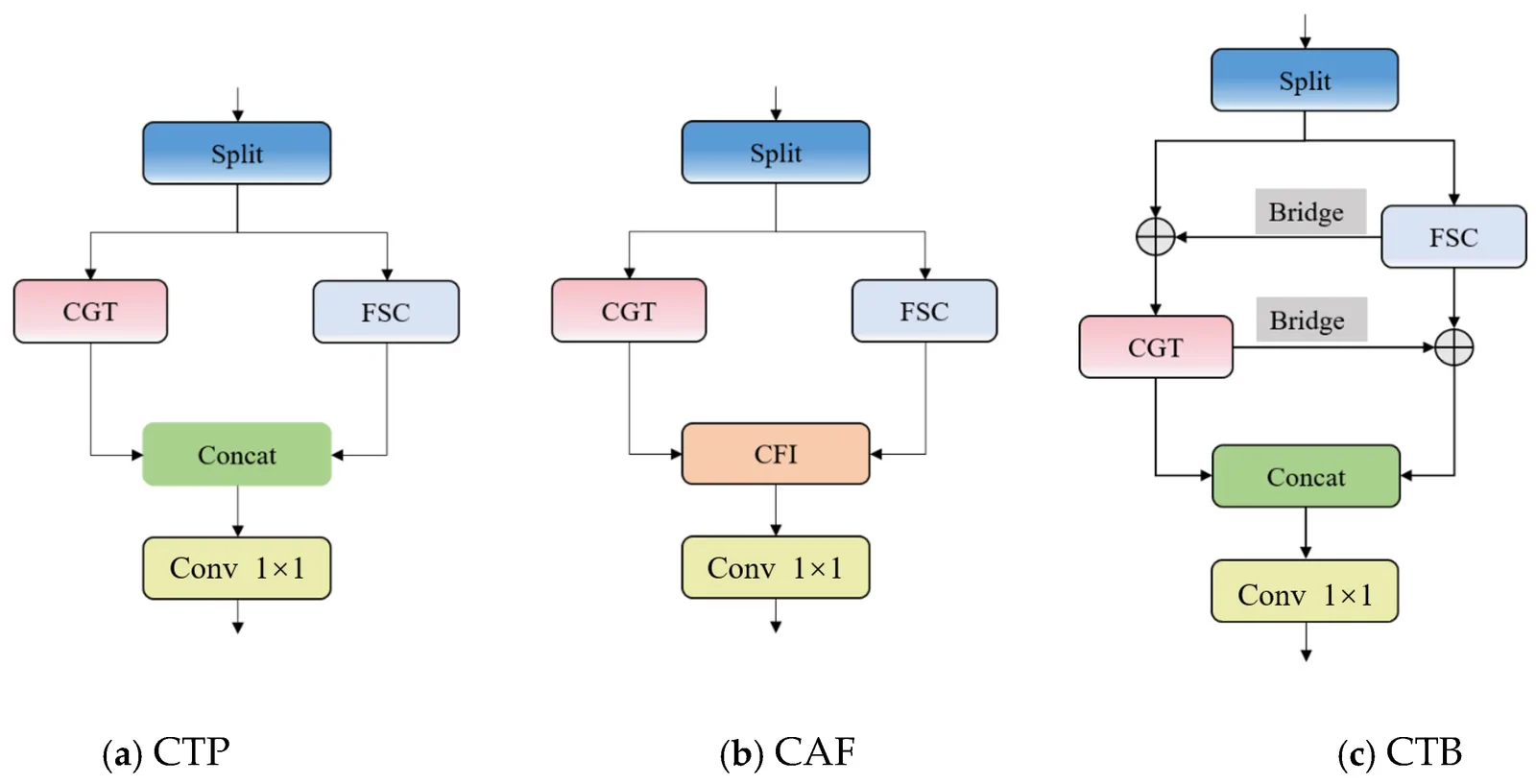
Comparison of bridging methods: (**a**) CTP without cross-branch exchange; (**b**) CAF with cross-attention fusion; and (**c**) the proposed CTB. Each Bridge in (**c**) is a learned 3 × 3 projection to the receiving branch; the arrows show the CNN-to-Transformer and Transformer-to-CNN directions, followed by concatenation and 1 × 1 output fusion.

A direct parallel control, denoted CTP, splits the input between CGT and FSC and concatenates their outputs without cross-branch exchange, as shown in Figure 6a. This control isolates the effect of interaction from the effect of simply using two branches.

A stronger comparison baseline, Cross-Attention Fusion (CAF), introduces a cross-feature interaction operation before output fusion, as shown in Figure 6b. CAF increases parameter count and computational cost relative to direct concatenation. The abbreviation CAF is used consistently here and in the ablation study in Section 3.2.3.

The proposed CTB is shown in Figure 6c. It performs one local-to-global transfer followed by one global-to-local return within each block rather than repeated iterative fusion. This two-direction pass was selected to keep the interaction path explicit and to bound additional computation and activation memory; the present study does not claim that it is universally superior to iterative fusion. Let X with shape B×C×H×W be split into $X_{c}$ with shape B×C×H×W and $X_{t}$ with shape B×bC×H×W, where a+b=1. $B_{t\to c}$ and $B_{c\to t}$ are learned 3 × 3 projections whose output channels match the receiving branch. Consistent with the asymmetric data flow in Figure 6c, the local input first informs CGT, and the resulting contextual feature is then returned to the FSC output before fusion:

$$\begin{array}{l} F=\mathrm{FSC}(X_{C}),T=\mathrm{CGT}(X_{T}+B_{C\to T}(X_{C}))\\ F_{\mathrm{out}}=F+B_{T\to C}(T),Y={\mathrm{Conv}}_{1\times 1}(\mathrm{Concat}(T,F_{\mathrm{out}})) \end{array}$$

Figure 6 represents each Bridge as a 3 × 3 channel projection; additional bridge-specific normalization or activation is not specified in the reported architecture. When $b=0,X_{t},T$, and both bridge projections are absent, and the stage reduces to FSC-only processing followed by the stage output projection.

### 2.4. Multi-Scale Feature Pyramid Network (MS-FPN)

This paper uses the established feature pyramid principle and redesigns the fusion pathway for the present defect segmentation backbone. The novelty claim is therefore restricted to the MSF-AHDown arrangement described below, not to multi-scale feature pyramids in general.

**Figure 7:**
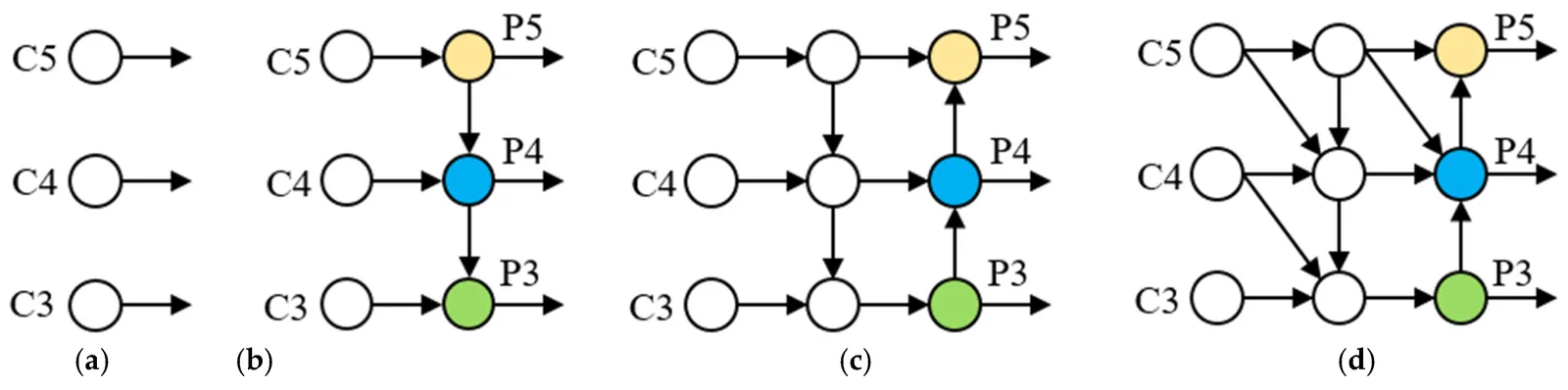
Comparison of feature fusion structures: (**a**) non-fusion; (**b**) single-direction fusion; (**c**) simple bidirectional fusion; and (**d**) complex bidirectional fusion.

As shown in Figure 7, several FPN variants have been developed. The non-fusion FPN is represented by SSD [46], as shown in Figure 7a. Top-down single-direction fusion is commonly used in Faster R-CNN [47], Mask R-CNN, and YOLOv3 [48], as shown in Figure 7b. PANet introduces an additional bottom-up path to form bidirectional fusion, as shown in Figure 7c. More complex bidirectional fusion structures, such as ASFF [49], NAS-FPN [50], and BiFPN [51], are shown in Figure 7d. However, in industrial inspection scenes with complex backgrounds and many interfering textures, overly complex multi-scale fusion may introduce noise and degrade performance [46,47,48,49,50,51].

**Figure 8:**
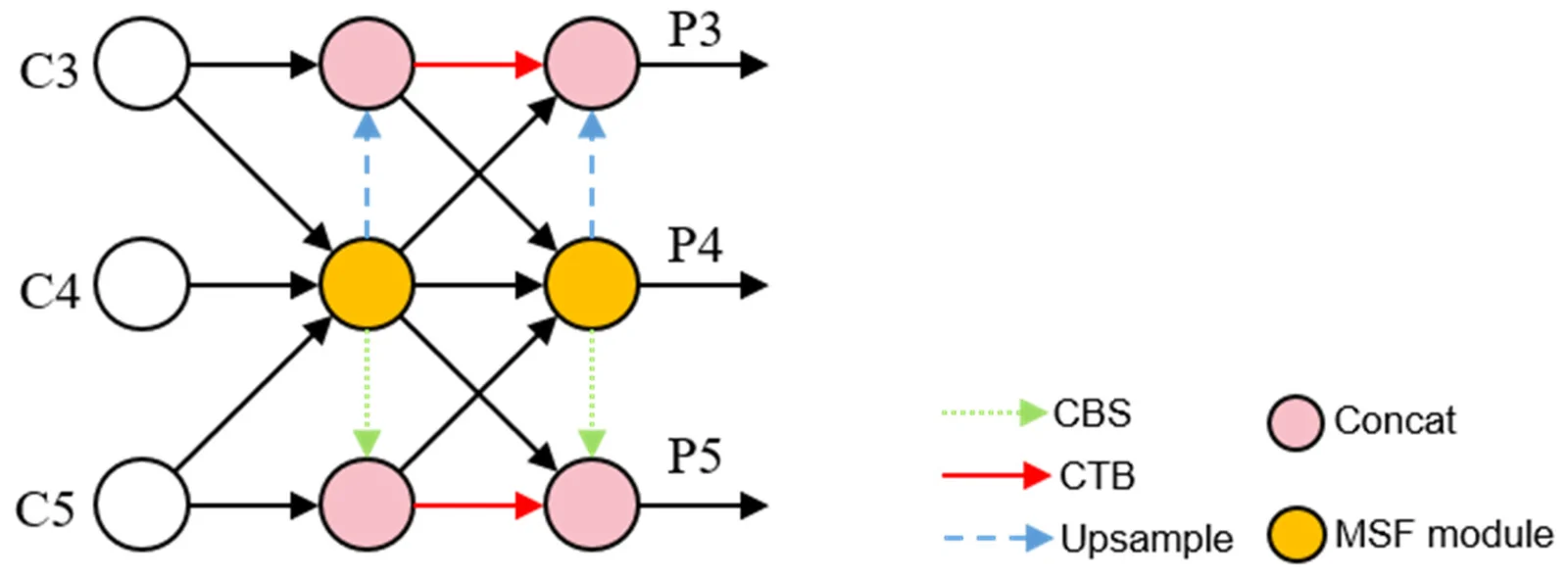
Overall architecture of MS-FPN. CBS denotes convolution, normalization, and activation operations.

**Figure 9:**
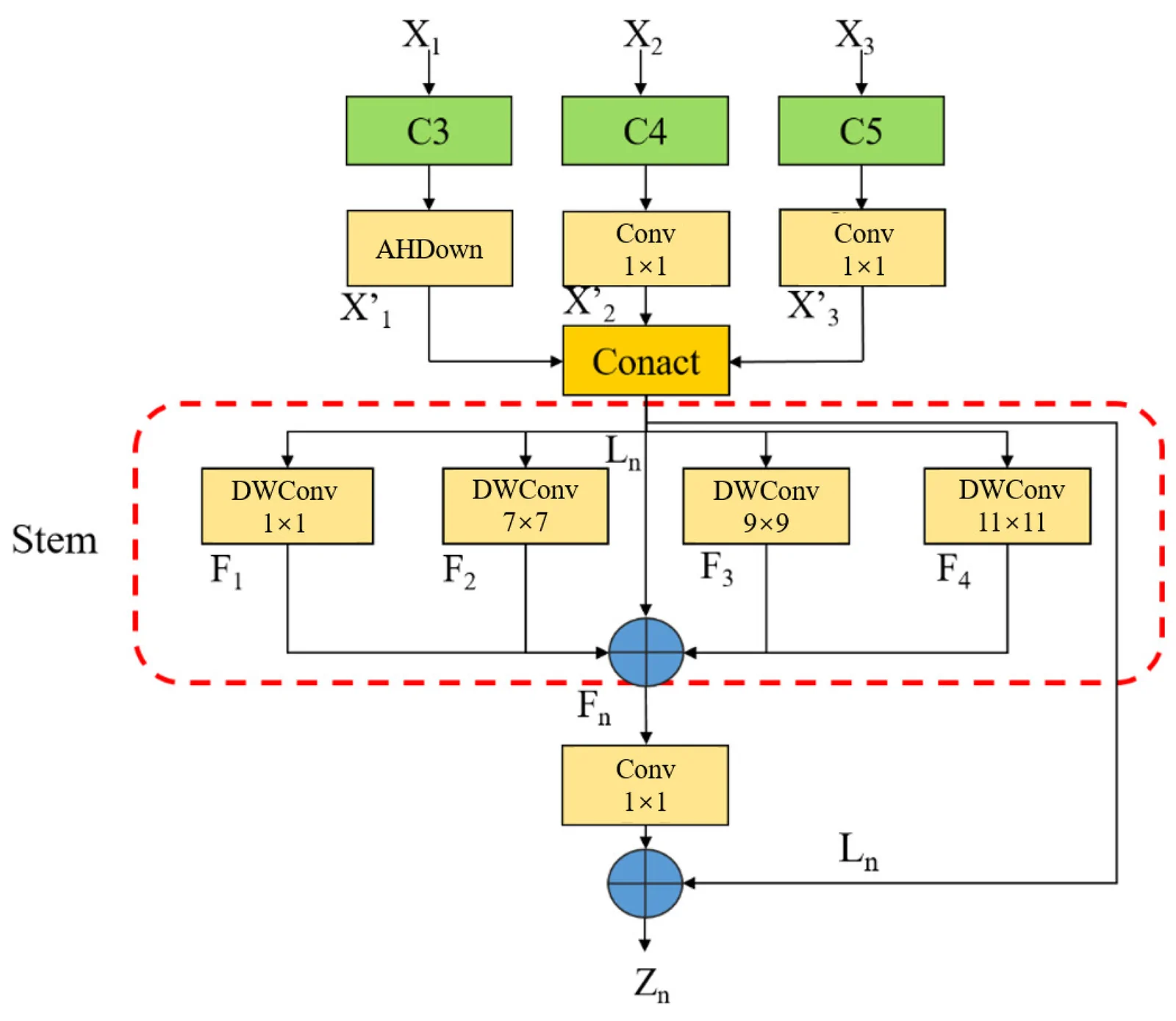
Overall architecture of the MSF module.

**Figure 10:**
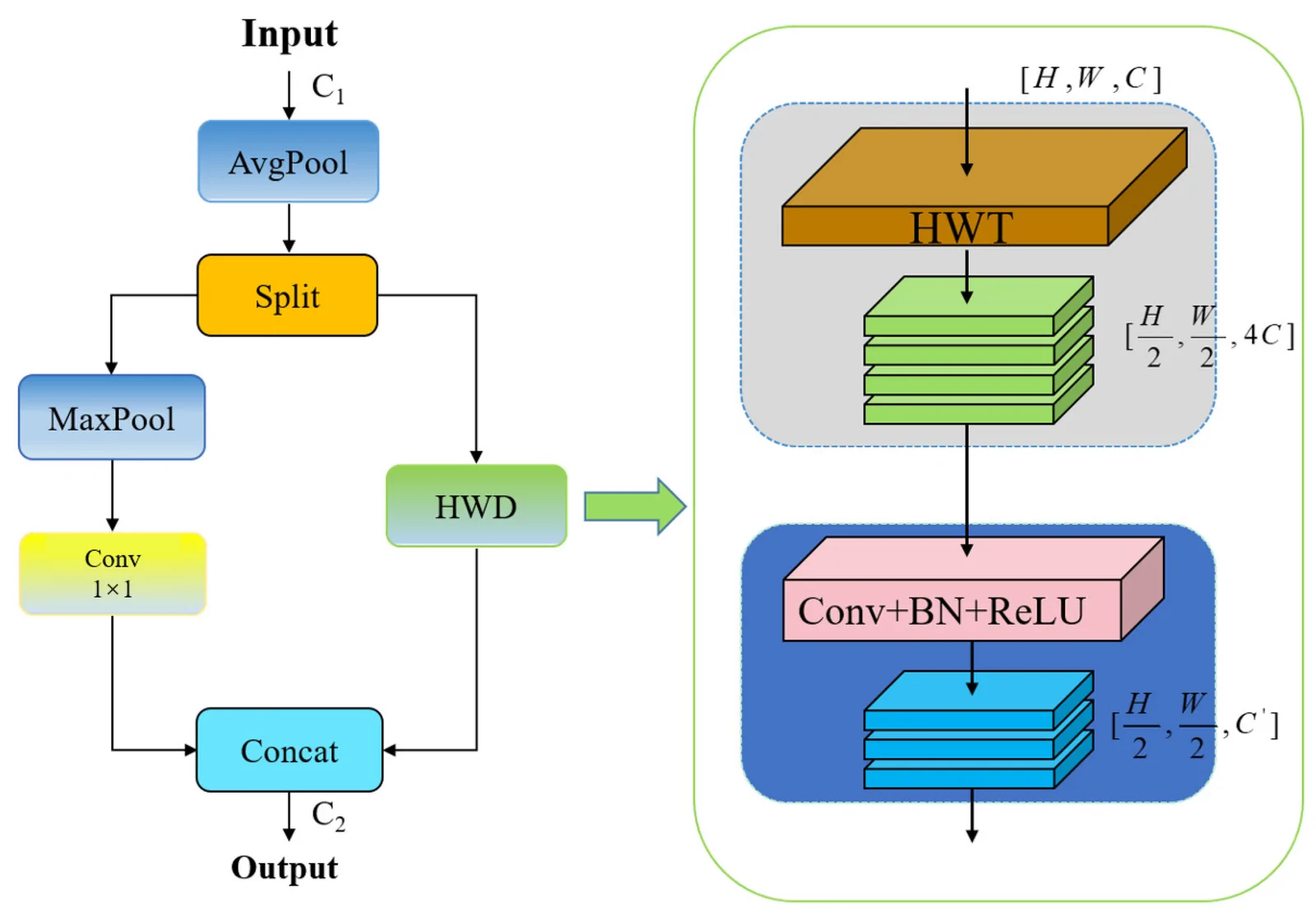
AHDown (AvgPool Haar Down) module.

Based on these observations, MS-FPN aligns feature maps C3, C4, and C5, concatenates the aligned maps, and processes the result with the MSF and AHDown modules shown in Figure 8, Figure 9 and Figure 10. The task-specific design is intended to retain fine responses for small defects while adding broader context for larger defects; the present experiments do not isolate every classic FPN variant under a common protocol.

The MSF module is shown in Figure 9. Four centered odd-sized convolution kernels (5 × 5, 7 × 7, 9 × 9, and 11 × 11) provide progressively larger local receptive fields without the sparse sampling pattern of dilated convolution. The resulting features are followed by parallel depthwise convolutions, pointwise projection, and residual fusion.

After spatial and channel alignment, the C3-C5 features are concatenated to form L_n_, as shown in Figure 9 and Equation (11). Within the MSF stem, the four parallel depthwise-convolution responses F_i_ are then fused by element-wise summation in Equation (12); no learned scalar weighting is used in this operation. The term weighted combination is therefore not used for MSF.

The MSF module extracts features from C3, C4, and C5 and uses corresponding downsampling modules to align spatial resolution and channel dimensions. AHDown is adopted for downsampling because it preserves more useful information than standard downsampling while reducing feature-map resolution. After multi-scale fusion, parallel depthwise convolutions are used to extract contextual information. The process is expressed as follows: $X_{\left(1\right)}\in {\mathbb{R}}^{C_{1}\times H_{1}\times W_{1}},X_{\left(2\right)}\in {\mathbb{R}}^{C_{2}\times H_{2}\times W_{2}},X_{\left(3\right)}\in {\mathbb{R}}^{C_{3}\times H_{3}\times W_{3}}$. $L_{n}\in {\mathbb{R}}^{3C\times H^{\prime }\times W^{\prime }}$ is obtained. This is followed by parallel depthwise convolutions to extract contextual information from multiple scales. Mathematically, this process is expressed as follows:

$$X_{n}^{\prime }=\Delta (X_{n}),n\in 1,2,3$$

(11) $$L_{n}=Concat(X_{\left(1\right)}^{\prime },X_{\left(2\right)}^{\prime },X_{\left(3\right)}^{\prime })$$

After spatial-resolution and channel-dimension alignment, the three input features $X_{1}$, $X_{2}$, and $X_{3}$ are transformed into $X_{1}^{\prime }$, $X_{2}^{\prime }$, and $X_{3}^{\prime }$, respectively, and then concatenated to obtain the fused feature $L_{n}$, as expressed in Equation (11). Here, Δ denotes the feature-alignment operation. For n=1, the AHDown module is employed to downsample and align the feature map, whereas for n=2 and n=3, a 1×1 convolution is used to adjust the channel dimensions. 

Finally, the multi-scale contextual features are aggregated by element-wise summation and further fused with the original concatenated feature through a pointwise 1×1 convolution and a residual connection. The pointwise convolution integrates the multi-scale responses and compresses the channel representation, while the residual connection preserves the original local information and facilitates stable feature propagation. The final output $Z_{n}$ is calculated according to Equation (12).

(12) $$Z_{n}=L_{n}+\mathrm{Conv}(L_{n}+\sum_{i=1}^{4}F_{i})$$

In the MSF module, an average-pooling wavelet downsampling module, AvgPool Haar Down (AHDown), is proposed based on HDown, as shown in Figure 10. Average pooling first extracts representative information and reduces noise. The feature map is then split into two branches: one branch uses Haar wavelet transform for downsampling, and the other uses max pooling and convolution to preserve salient edge and detail information. The outputs are concatenated to fuse complementary downsampling information, reducing feature loss when resolution decreases [52].

### 2.5. HBCTNet Architecture

Following common backbone scaling strategies, three variants of HBCTNet are constructed: HBCTNet-S, HBCTNet-M, and HBCTNet-L. The number of stage-template blocks is adjusted according to model depth. Table 1 reports the configurations, where c denotes output channels and S, M, and L denote small, medium, and large variants, respectively. In this table, CTB denotes the stage template; when the Transformer coefficient b is zero, the template reduces to an FSC-only block.

### 2.6. Dataset

The dataset used in this study comprises industrial images acquired from a piezoelectric crystal production line. Pixel-level instance masks were created manually with Labelme. The evaluation treats the masks as aggregate defect instances; verified subtype labels and per-subtype instance counts are not available in the reported record. Camera resolution and settings, physical-crystal identifiers, production-batch identifiers, and illumination strata are also not reported. Accordingly, no per-class, crystal-independent, cross-batch, or illumination-specific performance claim is made.

A total of 3298 annotated images were used. The dataset was divided at image level into 2638 training images, 330 validation images, and 330 test images, corresponding approximately to an 80/10/10 split. Training-time augmentation is described separately in Section 3.1 and does not alter these nominal split counts. The available description does not state whether physical-crystal or production-batch identifiers were used when forming the subsets, or whether the partition was stratified by illumination or defect subtype. Residual correlation and class imbalance therefore cannot be quantified from the present record.

**Figure 11:**
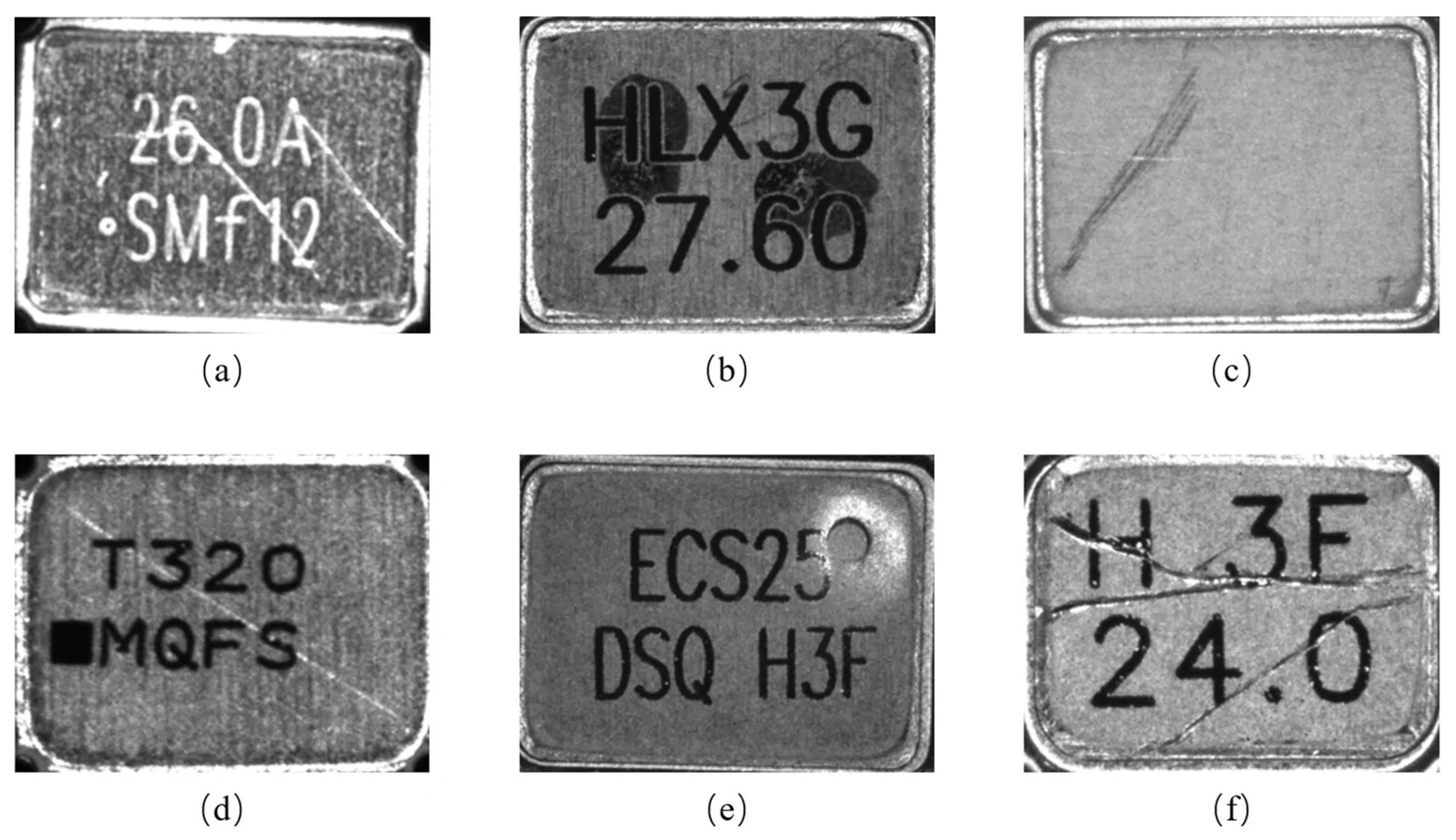
Raw examples from the piezoelectric crystal dataset: (**a**) printed marking with a diagonal scratch; (**b**) high-contrast printed marking with a localized surface spot; (**c**) an elongated scratch on a low-texture surface; (**d**) printed marking with a diagonal scratch; (**e**) printed marking with a circular stain-like defect; and (**f**) multiple line/scratch defects. The examples illustrate variation in printed markings, surface texture, contrast, and defect morphology. Ground-truth mask overlays are not shown in this figure.

The available annotation protocol documents one manually produced reference-mask set but does not document annotator professional background, independent double annotation, inter-annotator agreement, or formal adjudication. Annotation uncertainty is therefore not quantified and should be considered when interpreting pixel-level performance. Representative raw samples are shown in Figure 11.

## 3. Experimental Results

### 3.1. Implementation Details

HBCTNet and its ablation variants were trained using stochastic gradient descent (SGD) with momentum of 0.937 and weight decay of 0. A three-epoch linear warm-up was used with initial warm-up momentum of 0.8 and initial learning rate of 0.01. Mask overlap was allowed, the mask downsampling ratio was 4, and training-time augmentation included cropping, flipping, translation, saturation adjustment, and mosaic augmentation. These models were trained for 300 epochs without pretrained weights. Weight decay 0 was a fixed SGD configuration shared by the HBCTNet ablations; no dedicated weight-decay ablation is reported, and the setting is not claimed to be optimal.

The settings above apply to HBCTNet and its within-model component ablations. For the cross-model comparison in Section 3.3, baseline-specific optimizer, loss, and augmentation configurations are not documented. The stated methods therefore do not establish whether every baseline used an identical training pipeline or its separately optimized default pipeline, which limits reproducibility and interpretation of augmentation fairness.

Input resolution, batch size, the complete loss terms and weights, the learning-rate schedule after warm-up, random seed, software versions, hardware specification, checkpoint-selection rule, and training time are not specified in the reported methods. These omissions limit exact reproduction and prevent a verified analysis of run-to-run variability.

### 3.2. Ablation Studies

#### 3.2.1. Effect of the Frequency and Spatial Convolution Block

To evaluate the effectiveness of FSC, this ablation study replaces the FSC module in HBCTNet with the Ghost module from GhostNet and the PConv module from FasterNet. All other modules and parameter settings are kept unchanged for a fair comparison.

As shown in Table 2, FSC achieves the highest mask AP among the compared convolution blocks.

#### 3.2.2. Effect of the Number of Attention Heads

The number of attention heads determines both the number of attention subspaces and the channel dimension assigned to each head. The active Transformer-channel counts in the deeper stages are divisible by eight, so h=8 provides an integer head dimension while retaining multiple subspaces. Four head configurations were compared to evaluate the empirical effect of this choice.

Table 3 shows the reported mask AP for each configuration. Eight heads obtain the highest reported value and are used in the remaining experiments. This is an empirical, architecture-dependent choice rather than a universal theoretical optimum.

#### 3.2.3. Effect of Convolution-Transformer Bidirectional Bridging Blocks

As described in Section 2.3, CTP, CAF, and CTB were compared under the same branch setting. Table 4 shows that CTB obtains the highest reported mask AP with comparable parameter count and FLOPs. The result supports the selected interaction design on this dataset, but it is not a statistical robustness test.

#### 3.2.4. Effect of Branch Weight Coefficients

The stage template allows the channel proportions a and b of the FSC and CGT branches to be adjusted. When b=0, the CGT branch and both cross-branch projections are omitted and the stage reduces to FSC-only processing. Thus, a stage labeled with the CTB template does not necessarily contain an active Transformer branch.

As shown in Table 5, the highest reported mask AP is obtained with Transformer coefficients 0, 0, 0.25, and 0.5 from Stages 1 to 4. The result favors local processing in the high-resolution early stages and introduces global attention only after spatial resolution has decreased.

In Table 5, each pair (a, b) denotes the channel proportions of the FSC and CGT branches, respectively. The (1, 0) setting means FSC-only processing, not bidirectional CNN–Transformer interaction.

#### 3.2.5. Effect of Kernel Number and Multi-Scale Kernel Design

The number of parallel convolution paths in the MSF stem was first evaluated. As shown in Table 6, four paths obtain the highest reported mask AP among the tested path counts.

After fixing four paths, centered odd-sized kernel combinations with a step of two were evaluated. The 5 × 5, 7 × 7, 9 × 9, and 11 × 11 combination provides receptive-field radii from 2 to 5 pixels at the feature-map scale while preserving dense sampling. Table 7 shows that this combination gives the highest reported mask AP among the tested sets. The result is dataset- and architecture-dependent and is not presented as a universal theoretical optimum.

### 3.3. Comparison with State-of-the-Art Models

To evaluate HBCTNet on the same dataset, it was compared with representative instance segmentation models in Table 8. The reported experiment description states that the same data split and hardware/software platform were used. However, baseline-specific optimizer, loss, and augmentation configurations are not documented, so it cannot be determined from the stated methods whether every model used an identical pipeline or a separately optimized default pipeline. Table 8 is therefore interpreted as a cross-model point-estimate comparison rather than proof of either a strictly controlled training comparison or the maximum performance of separately optimized baselines.

In the reported comparison, the HBCTNet variants achieve higher box AP and mask AP than the listed baselines. These values describe performance on the present image-level split; without repeated runs or an external dataset, they do not establish statistical significance, cross-domain generalization, or deployment robustness.

### 3.4. Qualitative Visualization

**Figure 12:**
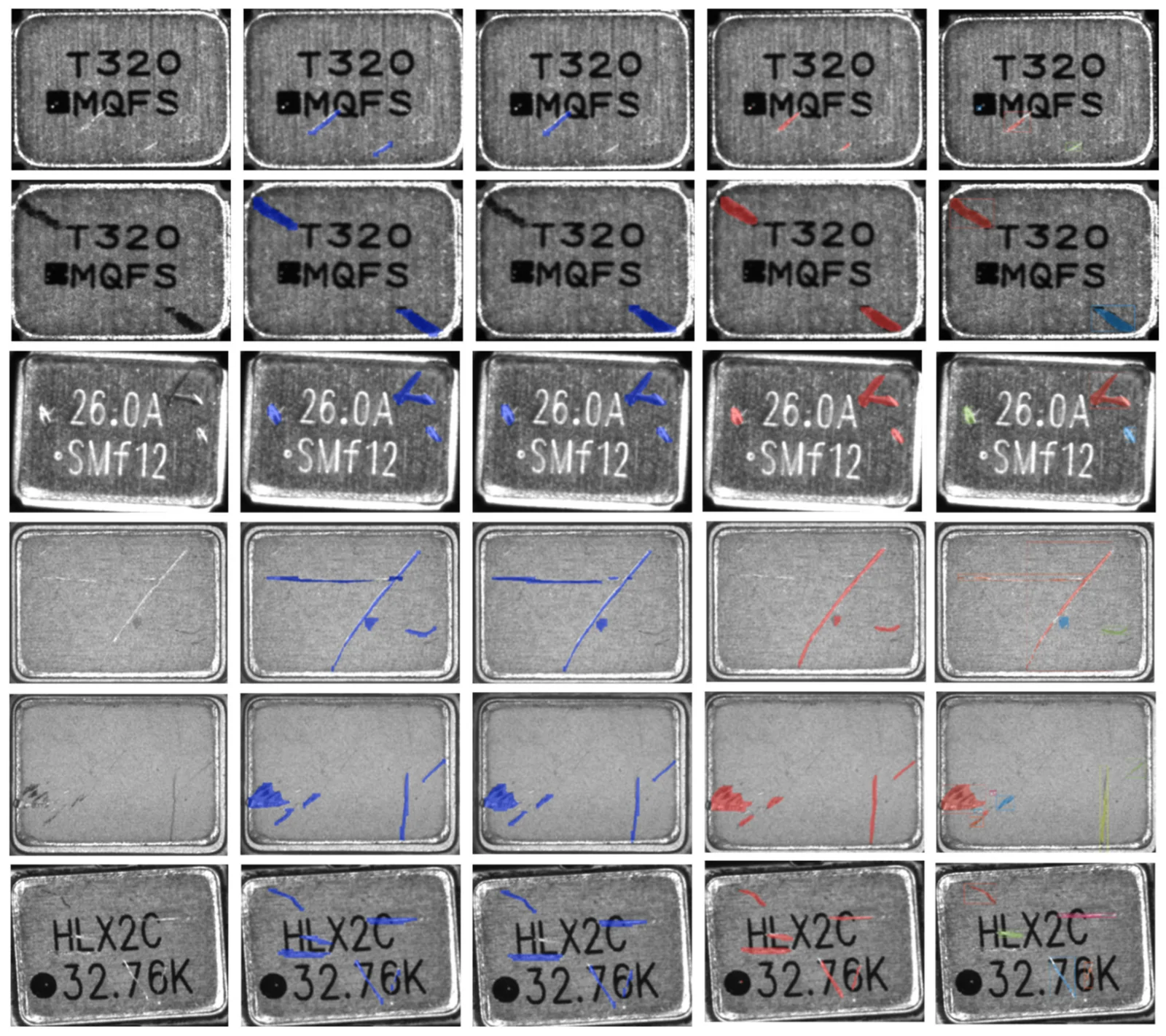
Representative qualitative instance segmentation outputs from the compared algorithms. Colored contours indicate predicted instance boundaries for visual comparison. Aggregate box AP and mask AP are reported in Table 8; this figure is not a separate quantitative evaluation.

Figure 12 qualitatively illustrates representative predictions from HBCTNet, YOLO11-SEG, YOLOv9-SEG, and YOLACT. In the shown examples, the baseline outputs contain missed or incomplete regions for some large, low-contrast, or finely textured defects, whereas HBCTNet retains more of the visible defect regions. The aggregate quantitative comparison is provided by box AP and mask AP in Table 8. The visual observations are illustrative and are not additional per-image IoU, Dice, or F1 measurements.

## 4. Discussion

Piezoelectric crystal production can generate defects with substantial variation in size, type, distribution, and morphology, including scratches, oil stains, and small structural defects. Some defects show textures similar to the normal crystal surface, making them difficult to identify. These characteristics create significant challenges for industrial defect segmentation.

HBCTNet combines local and global feature modeling in the deeper backbone stages. CTB performs one reciprocal exchange between FSC and CGT features, FSC extracts spatial- and frequency-domain responses, and MS-FPN aligns and aggregates the last three feature scales. The ablations in Table 2, Table 3, Table 4, Table 5, Table 6 and Table 7 provide component-level evidence for selected alternatives under the reported point-estimate protocol.

On the reported image partition, HBCTNet-S achieves 92.3% box AP and 89.9% mask AP. Its mask AP is 2.1 percentage points higher than YOLO11-SEG and 2.4 percentage points higher than YOLOv9-SEG. Table 2 compares FSC with Ghost and PConv blocks, and Table 4 compares CTP, CAF, and CTB. These are descriptive point estimates and do not constitute a statistical significance analysis.

The evidence has several limitations. Evaluation is confined to one production-line dataset and a reported 80/10/10 image partition without verified crystal, batch, illumination, or defect-subtype strata. Per-class distribution, independent annotation agreement, external-dataset performance, repeated-run variability, confidence intervals, and formal significance tests are not available. The ablations do not isolate the spatial and frequency branches separately, do not compare all classic FPN variants, do not test iterative fusion, and do not provide a cumulative add-one-component analysis or intermediate feature maps. Consequently, claims are restricted to the reported within-dataset results.

Potential failure modes include extremely weak-contrast defects, defect boundaries confused with printed markings or normal surface texture, and acquisition conditions outside the training distribution. The current study does not provide a systematically categorized failure set, so these scenarios remain qualitative limitations rather than measured error rates.

Deployment feasibility is also unresolved. HBCTNet-S requires 48.1 G FLOPs, compared with 35.3 G for YOLO11-SEG and 42.4 G for YOLOv8s-SEG in Table 8. FLOPs do not determine end-to-end latency, and no FPS, inference-time, GPU-memory, CPU-throughput, or production-line timing measurements are reported. Real-time operation is therefore a future validation target rather than a conclusion of the present study.

## 5. Conclusions

This study proposes HBCTNet for instance segmentation of piezoelectric crystal defects. In the selected configuration, FSC-only early stages preserve local detail, deeper CTB stages exchange FSC and CGT features, and MS-FPN aggregates multi-scale features through MSF and AHDown. On the reported image partition, HBCTNet-S obtains 92.3% box AP and 89.9% mask AP with 12.67 M parameters and 48.1 G FLOPs. These results support the architecture within the evaluated dataset; they do not constitute physical defect metrology, cross-domain validation, statistical significance, or real-time deployment evidence.

Future work should establish crystal- and batch-independent splits, per-defect statistics, independent annotation quality control, repeated-seed confidence intervals, public-dataset transfer, cumulative component ablations, and systematic failure-case analysis. Deployment studies should report end-to-end latency, FPS, GPU memory, CPU throughput, and the complete preprocessing and postprocessing pipeline before real-time industrial use is claimed.

## Figures and Tables

**Table 1:** Configurations of HBCTNet-S/M/L.

Stage	Output Size	Layer Name	HBCTNet-S	HBCTNet-M	HBCTNet-L
Stem	$\frac{H}{2}\times \frac{W}{2}$	Convolution Layer	[Conv 3 × 3] c = 32; s = 2	[Conv 3 × 3] c = 48; s = 2	[Conv 3 × 3] c = 64; s = 2
Stage1	$\frac{H}{4}\times \frac{W}{4}$	Convolution Layer	Conv 3 × 3; c = 64; s = 2	Conv 3 × 3; c = 96; s = 2	Conv 3 × 3; c = 128; s = 2
		CTB	CTB × 1, 64	CTB × 2, 96	CTB × 3, 128
Stage2	$\frac{H}{8}\times \frac{W}{8}$	Convolution Layer	[Conv 3 × 3] c = 128; s = 2	[Conv 3 × 3] c = 192; s = 2	[Conv 3 × 3] c = 256; s = 2
		CTB	CTB × 2, 128	CTB × 4, 192	CTB × 6, 256
Stage3	$\frac{H}{16}\times \frac{W}{16}$	Convolution Layer	[Conv 3 × 3] c = 256; s = 2	[Conv 3 × 3] c = 384; s = 2	[Conv 3 × 3] c = 512; s = 2
		CTB	CTB × 2, 256	CTB × 4, 384	CTB × 6, 512
Stage4	$\frac{H}{32}\times \frac{W}{32}$	Convolution Layer	[Conv 3 × 3] c = 512; s = 2	[Conv 3 × 3] c = 576; s = 2	[Conv 3 × 3] c = 512; s = 2
		CTB	CTB × 1, 512	CTB × 2, 576	CTB × 3, 512
Params			12.67 M	31.89 M	60.94 M
FLOPs			48.1 G	125 G	254.5 G

**Table 2:** Comparison of different convolution blocks. Bold values indicate the best result in the corresponding comparison.

Module	Params	FLOPs	AP_mask_
Ghost Block	12.35 M	47.5 G	88.1%
PConv Block	12.30 M	47.3 G	87.7%
**FSC**	12.67 M	48.1 G	**89.9%**

**Table 3:** Comparison of different numbers of attention heads. Bold values indicate the best result in the corresponding comparison.

Head	Params	FLOPs	AP_mask_
1	12.67 M	48.4 G	88.5%
2	12.67 M	48.1 G	88.7%
4	12.67 M	48.1 G	88.9%
**8**	12.67 M	48.1 G	**89.9%**

**Table 4:** Comparison of different bridging methods. Bold values indicate the best result in the corresponding comparison.

Method	Params	FLOPs	AP_mask_
CTP	12.63 M	48.0 G	87.8%
CAF	12.92 M	48.6 G	88.2%
**CTB**	12.67 M	48.1 G	**89.9%**

**Table 5:** Comparison of different branch weight coefficients. Bold values indicate the best result in the corresponding comparison.

Stage1	Stage2	Stage3	Stage4	Params	FLOPs	AP_mask_
(1, 0)	(1, 0)	(0.75, 0.25)	(0.5, 0.5)	12.67 M	48.1 G	**89.9%**
(1, 0)	(0.75, 0.25)	(0.75, 0.25)	(0.5, 0.5)	12.89 M	49.8 G	88.7%
(1, 0)	(0.75, 0.25)	(0.5, 0.5)	(0.25, 0.75)	11.30 M	47.7 G	87.6%
(1, 0)	(0.5, 0.5)	(0.5, 0.5)	(0.25, 0.75)	11.12 M	45.7 G	87.1%

**Table 6:** Comparison of different kernel numbers. Bold values indicate the best result in the corresponding comparison.

Number	Params	FLOPs	AP_mask_
3	12.56 M	47.7 G	88.8%
**4**	12.67 M	48.1 G	**89.8%**
5	12.84 M	48.6 G	88.9%

**Table 7:** Comparison of different multi-scale kernel designs. Bold values indicate the best result in the corresponding comparison.

Kernel	Params	FLOPs	AP_mask_
(1, 3, 5, 7)	12.49 M	47.5 G	88.0%
(3, 5, 7, 9)	12.57 M	47.7 G	87.9%
**(5, 7, 9, 11)**	12.67 M	48.1 G	**89.9%**
(7, 9, 11, 13)	12.81 M	48.5 G	87.1%

**Table 8:** Comparison with state-of-the-art models. Bold values indicate the best result in the corresponding comparison.

Model	Params	FLOPs	AP_box_	AP_mask_
YOLOv5s-SEG	7.4 M	25.7 G	87.4%	86%
YOLOv7-SEG	27.8 M	141.9 G	91%	87%
YOLOv8s-SEG	11.77 M	42.4 G	91.2%	86.7%
YOLOv9-SEG	27.4 M	145.5 G	90.3%	87.5%
YOLO11-SEG	10.07 M	35.3 G	91.5%	87.8%
**HBCTNet-S**	12.67 M	48.1 G	**92.3%**	**89.9%**
Mask R-CNN	45.8 M	104.2 G	79.6%	75.2%
CenterMask2	34.5 M	112.6 G	78%	76.1%
TensorMask	44 M	179.6 G	-	64.9%
RetinaMask	38 M	130.5 G	76.3%	72.5%
ShapeMask	50 M	189.1 G	68.9%	65.2%
YOLACT	33.5 M	111.7 G	65.2%	58.4%
YOLACT++	34.5 M	115.3 G	72.5%	65.1%
**HBCTNet-M**	31.87 M	125.0 G	**93.3%**	**91.0%**
**HBCTNet-L**	60.90 M	253.7 G	**95.2%**	**93.1%**

## Data Availability

The data presented in this study are available from the corresponding author upon reasonable request.
